# Direct and indirect effects of psychological well-being and therapeutic alliance on therapy outcome in eating disorders

**DOI:** 10.3389/fpsyg.2024.1392887

**Published:** 2024-05-24

**Authors:** Laura Muzi, Nicola Carone, Marta Mirabella, Anna Franco, Michele A. Rugo, Claudia Mazzeschi, Vittorio Lingiardi

**Affiliations:** ^1^Department of Philosophy, Social Sciences, Humanities and Education, University of Perugia, Perugia, Italy; ^2^Department of Systems Medicine, University of Rome Tor Vergata, Rome, Italy; ^3^Department of Dynamic and Clinical Psychology, and Health Studies, Sapienza University of Rome, Rome, Italy; ^4^Eating Disorder Clinic “Residenza Gruber”, Bologna, Italy

**Keywords:** psychological well-being, therapeutic alliance, anorexia nervosa, bulimia nervosa, residential treatments, therapy outcome

## Abstract

**Introduction:**

Outcome research in eating disorders (EDs) is commonly focused on psychopathological dysfunction. However, Ryff’s model of psychological well-being (PWB) has shown promising—yet preliminary—results with ED patients. Additionally, despite substantial evidence highlighting the association between the therapeutic alliance and treatment outcome, findings in ED samples remain unclear. The present study aimed at exploring the direct effect of PWB dimensions and the early therapeutic alliance on ED patients’ individual treatment responses, as well as the mediating role played by the early therapeutic alliance in the relationship between PWB dimensions and overall pre-post symptom change.

**Methods:**

A sample of *N* = 165 ED patients assigned female at birth, who were receiving treatment in a residential program, completed the Psychological Well-Being Scale at treatment intake and the Working Alliance Inventory after the first four psychotherapy sessions. Patients also completed the Outcome Questionnaire-45.2 at the same time point and during the week prior to discharge.

**Results:**

The PWB dimensions of autonomy, positive relations, and self-acceptance were associated with clinically significant change, while the dimensions of personal growth and self-acceptance were associated with reliable change. The early therapeutic alliance showed both direct and indirect effects on therapy outcome, predicting clinically significant and reliable symptom reduction. It also emerged as a significant mediator in the relationship between all PWB dimensions and overall symptomatic change.

**Conclusion:**

The identification of individual, adaptive characteristics in ED patients that might influence their development of an early therapeutic alliance may help therapists to predict relationship ruptures and tailor their interventions to enhance treatment effectiveness.

## 1 Introduction

Eating disorders (EDs) are severe pathologies characterized by persistent disturbances in eating or weight control behaviors, as well as overconcern with weight and shape (Hilbert et al., 2017; Treasure et al., 2022). Due to their high complexity and tendency towards chronicity, they represent major areas of concern for clinicians, families, and society. EDs not only significantly impact patients’ health, psychosocial functioning, and quality of life, but they also incur large costs for the healthcare system (Ágh et al., 2016). Despite ongoing progress in the development of ED treatment, data suggest that outcomes across diagnoses remain less than optimal. For instance, recent meta-analytic reviews (e.g., Solmi et al., 2024) have underlined high dropout rates and limited outcomes, especially for patients at lower weights, as well as high mortality, low recovery rates, and a significant rate of relapse or hospitalization, even after periods of significant symptomatic remission.

The severity and chronicity of EDs might explain the significantly greater focus on psychopathological symptoms over adaptive and positive functioning in the literature (de Vos et al., 2018). However, as the World Health Organization has described since 1948, mental health is more than simply the absence of mental disorder or pathological symptoms (World Health Organization, 2022). Rather, mental health exists on a continuum and is also influenced by protective factors, including well-being (Vaillant, 2012; Lingiardi and McWilliams, 2017).

One of the most rigorously tested theoretically grounded models of *psychological well-being* (PWB) was introduced by Ryff (1989) and Ryff and Keyes (1995), whose framework refers to reliable indicators of positive functioning based on clinical and personality theories. In response to criticism of *subjective well-being* indicators (e.g., positive affect, life satisfaction) for ignoring key components of positive psychological functioning (Ryff and Singer, 2006), Ryff’s PWB model evaluates how individuals function psychologically in response to life demands, based on the *eudaimonic* notion that well-being derives from the realization of human potential (Ryan and Deci, 2001). In more detail, PWB is a multidimensional concept comprised of six dimensions: (a) self-acceptance, referring to an awareness and acceptance of one’s strengths and weaknesses; (b) positive relations, referring to deep and meaningful relationships with others; (c) personal growth, referring to progress as a result of the development of one’s strengths; (d) autonomy, referring to self-determination and independent decision-making; (e) environmental mastery, referring to the management of one’s personal circumstances to take advantage of opportunities; and (f) purpose in life, referring to the establishment and directed pursuit of personal goals. This conceptualization has also been operationalized into a structured, self-report instrument (Ryff and Keyes, 1995), which has found support on the basis of six evidential sources: factor validity, psychological correlates, cross-time dynamics, sociodemographic correlates, biological correlates, and therapeutic intervention efficacy (Ryff and Singer, 2006; Ryff, 2014; Browne et al., 2017; Blasco-Belled and Alsinet, 2022; van Dierendonck and Lam, 2023).

Within the field of EDs, most research has focused on health-related dimensions such as quality of life or subjective well-being (de la Rie et al., 2005; Doll et al., 2005; Jenkins et al., 2011; Mond et al., 2012). However, two relevant studies have explored PWB among ED outpatients (Tomba et al., 2014, 2017). The first study found that ED patients—and especially those with bulimia nervosa (BN)—showed more severe impairment in PWB dimensions compared to a healthy control group, and that most PWB dimensions were negatively related to ED symptom severity (Tomba et al., 2014). A subsequent study on a sample of patients with mixed ED diagnoses found improvement in all PWB domains following the completion of an ED-specialized outpatient treatment program (Tomba et al., 2017). These findings suggest the relevance of assessing PWB in ED samples, in addition to the standard measurement of Body Mass Index (BMI), ED-related symptomatology, and behavioral parameters. Furthermore, other studies, despite not specifically related to Ryff’s PWB model, have found that ED patients tend to adopt maladaptive interpersonal behaviors to regulate emotions, avoid confrontation, and manage negative experiences (Mason et al., 2022). ED patients have also been shown to suffer in the areas of self-directedness, self-awareness, and self-understanding, and to struggle with finding “meaning in life” (i.e., establishing a sense of personal coherence, purpose, and significance) (Marco et al., 2019). Although these variables have been found to influence ED treatment response (e.g., Jones et al., 2015; Muzi et al., 2021; Mirabella et al., 2023), evidence on the possible predictive role played by baseline PWB dimensions on therapy outcomes in this population are lacking.

Another widely debated variable in the ED field is the therapeutic alliance, defined as the collaborative relationship between patient and therapist, and comprised of three main elements: agreement on treatment goals, consensus on treatment tasks, and development of an affective bond (Bordin, 1979). Several meta-analyses have shown the therapeutic alliance to be a reliable predictor of positive treatment outcomes for various disorders, even after controlling for possible confounding variables (e.g., treatment setting, theoretical orientation), particularly in the early phases of psychotherapy (Flückiger et al., 2018; Del Re et al., 2021). However, the effect appears less clear for patients with EDs compared to those with other disorders, as quantitative research on the relationship between the therapeutic alliance and ED treatment outcome has yielded mixed results (Graves et al., 2017). Three meta-analytic studies have suggested that the therapeutic alliance is a central factor in the psychotherapy process and outcome for ED patients, given its apparent association with treatment completion, weight gain, decrease in depressive symptomology and overall ED symptoms, and faster recovery, especially in patients with anorexia nervosa (AN) (Zaitsoff et al., 2015; Graves et al., 2017; Werz et al., 2022). Furthermore, research has also suggested that the therapeutic alliance might mediate symptom reduction in psychotherapy, providing greater clarity regarding the processes by which treatment leads to change (Baier et al., 2020). On the other hand, some studies have found no direct or interactive relation between the therapeutic alliance and ED treatment outcome (e.g., Brown et al., 2013; Raykos et al., 2014).

Related to the therapeutic alliance, research has shown that clinicians tend to report negative perceptions, attitudes, and emotional responses towards their ED patients (e.g., Colli et al., 2015; Groth et al., 2020). A potential explanation for this is that ED patients’ struggle to maintain their body condition and their general difficulties in self-regulation may hinder the formation of a therapeutic alliance (Antoniou and Cooper, 2013). Additionally, the general egosyntonic nature of EDs may reinforce patients’ ambivalence towards change and disagreement regarding treatment goals and tasks (Lev Ari et al., 2024).

These observations underline the need for a deeper understanding of the role played by the therapeutic alliance in ED treatment, and its potential association with ED patient characteristics (Zaitsoff et al., 2015; Graves et al., 2017; Marzola et al., 2019). Nevertheless, to date, little is known about the individual factors that may contribute to (or hinder) the development of a strong therapeutic alliance with ED patients (Waller et al., 2012; Katznelson et al., 2020; Lev Ari et al., 2024). In this regard, several PWB dimensions may be explored as potential predictive factors for the therapeutic alliance with ED patients and the influence of the therapeutic alliance on ED treatment outcome (e.g., Prusiński, 2022). For instance, ED patients who experience empathic, satisfying, and trusting relationships with others may be more willing to establish a collaborative relationship with the therapist which, in turn, may positively affect their treatment response. Additionally, ED patients’ strong sense of mastery and perceived capacity to make effective use of opportunities in their environment, as well as greater self-acceptance and self-directedness, may foster shared agreement on treatment goals and tasks, helping them bear the ambivalent feelings towards symptom change that are demonstrated by many ED patients, thereby enhancing treatment compliance.

### 1.1 Present study

Consistent with the clinical and empirical literature referenced above, the present study aimed at addressing two main issues. First, it explored the direct effects of PWB dimensions and the early therapeutic alliance on ED patients’ responses to treatment. To address the impact of these variables on symptomatic change of each individual patient, the study applied the twofold criterion developed by Jacobson and Truax (1991). This criterion not only identifies patients who fail to improve during psychotherapy, but it also distinguishes between patients who improve to a level of normal functioning and those who improve significantly yet remain in a symptomatic range. Second, the research evaluated whether the early therapeutic alliance mediated the association between different PWB dimensions and treatment outcome, defined as the pre-post change in overall symptomatic impairment. Despite the limited research on this topic, we expected that baseline PWB dimensions—especially positive relations, self-acceptance, environmental mastery, and purpose in life (Tomba et al., 2014; Browne et al., 2017)—would predict clinically significant and reliable therapeutic change. Furthermore, despite the mixed findings on ED samples, we hypothesized that the early therapeutic alliance would significantly predict clinically significant and reliable symptom reduction (Zaitsoff et al., 2015; Graves et al., 2017). Furthermore, in line with studies showing an indirect effect of the therapeutic alliance on therapy outcome (Baier et al., 2020) and the potential relevance of ED patients’ individual characteristics in determining this association (Prusiński, 2022; Lev Ari et al., 2024), we expected that the therapeutic alliance would mediate the relationship between PWB dimensions and overall symptomatic change.

## 2 Materials and methods

### 2.1 Participants

Participants were patients who had been consecutively admitted to a specialized, intensive, and psychodynamic-oriented residential ED treatment center in Bologna (Italy) between September 2019 and June 2023. The inclusion criteria were: (a) at least 18 years of age; (b) a DSM-5/DSM-5-TR diagnosis of AN or BN, established at intake by the consensus of a licensed staff psychiatrist and a clinical psychologist, based on the Structured Clinical Interview for DSM-5 (SCID-5-CV; First et al., 2016); (c) assigned female at birth; and (d) lacking any organic syndrome, psychotic disorder, or syndrome with psychotic symptoms that could complicate the assessment of any study variable.

An initial sample of *N* = 187 patients met these criteria. Of these, five patients (2.7%) were excluded due to premature discharge or dropout and 17 (9.1%) were not considered due to missing data at treatment intake or discharge. Out of the final study sample of *N* = 165 patients who completed all assessment measures, 78 (47.3%) were diagnosed with AN-Restricting Type (AN-R), with an average baseline BMI of 15.46 kg/m^2^ (*SD* = 2.56); 26 (15.8%) met the diagnostic criteria for AN-Purging Type (AN-P), with an average baseline BMI of 16.71 kg/m^2^ (*SD* = 2.28); and 61 (37%) fulfilled the diagnostic criteria for BN, with an average BMI of 23.31 kg/m^2^ (*SD* = 2.82). Participants’ mean age was 22.7 years (*SD* = 6.90), and all were cisgender and White. Most patients were single or separated (*n* = 159, 96.4%) and had no previous instance of hospitalization in an ED specialized treatment center (*n* = 86, 58.9%). Their mean age of ED onset was 15.6 years (*SD* = 4.01). The majority of patients (*n* = 103, 67.3%) showed at least one comorbid DSM-5/DSM-5-TR clinical syndrome, as assessed by the SCID-5-CV. More specifically, 22 patients (13.3%) fulfilled the diagnostic criteria for major depressive disorder, 16 (9.7%) for obsessive-compulsive disorder, and 12 (7.2%) for a persistent depressive disorder. Other comorbid clinical syndromes were present in less than 5% of all patients. Additionally, 113 patients (72%) showed at least one comorbid DSM-5/DSM-5-TR personality disorder. Most patients met the diagnostic criteria for borderline personality disorder (*n* = 49, 29.7%) and obsessive-compulsive personality disorder (*n* = 41, 24.8%) as primary diagnoses, whereas a lower percentage of patients fulfilled the diagnostic criteria for avoidant personality disorder (*n* = 7, 4.2%), dependent personality disorder (*n* = 4, 2.4%), narcissistic personality disorder (*n* = 3, 1.8%), and schizoid personality disorder (*n* = 3, 1.8%). The remaining patients (*n* = 6, 3.6%) showed an other specified/unspecified personality disorder.

### 2.2 Measures

Sociodemographic and baseline clinical characteristics (e.g., age, marital status, length of stay in residential treatment, age of ED onset) were available from each patient’s clinical chart.

#### 2.2.1 Structured Clinical Interview for DSM-5, clinician version (SCID-5-CV)

The SCID-5-CV (First et al., 2016; see also Fossati and Borroni, 2017) is a semi-structured interview that is used to categorically assess psychopathology according to the DSM-5/DSM-5-TR. It is typically administered by a clinician who is familiar with the DSM diagnostic criteria. Interview questions are provided alongside each DSM criterion to aid users in rating each criterion as either present or absent. The SCID-5-CV has shown excellent reliability and high specificity, as assessed using different methods, as well as good clinical validity (Osório et al., 2019).

#### 2.2.2 Psychological Well-Being scale (PWB)

The Psychological Well-Being Scale (PWB; Ryff and Singer, 1996; see also Ruini et al., 2003) is an 84-item measure derived from Ryff’s (1989) theoretically grounded, multidimensional model of psychological well-being. It captures six PWB dimensions: autonomy, environmental mastery, personal growth, positive relations, purpose in life, and self-acceptance. Participants respond to each item using a 6-point Likert scale ranging from 1 (*strongly disagree*) to 6 (*strongly agree*). Responses to negatively formulated items are reversed scored, so that higher scores indicate a higher self-rating on the assessed dimension. Scores for each dimensional scale range from 14–84. Previous studies have provided support for the PWB’s good-to-excellent internal consistency and high test–retest reliability (Ryff, 1989, 2014). In the present sample, Cronbach’s alpha for the six dimensions were: 0.84 for autonomy, 0.77 for environmental mastery, 0.76 for personal growth, 0.85 for positive relations, 0.81 for purpose in life, and 0.73 for self-acceptance.

#### 2.2.3 Working Alliance Inventory-Patient Version (WAI-P)

The Working Alliance Inventory (WAI; Horvath and Greenberg, 1989; see also Lingiardi, 2002) is a widely used 36-item self-report questionnaire based on Bordin’s (1979) conceptualization of the therapeutic alliance. It consists of three subscales (i.e., Bond, Task, Goal) and also generates a total score. Each item is rated on a 7-point Likert scale ranging from 1 (*never*) to 7 (*always*). The inventory is comprised of a set of related measures that include client-, therapist-, and observer-rated versions. The present study utilized the patient-rated version and the WAI overall score. A large body of research supports the reliability (Hanson et al., 2002), construct validity (e.g., Tichenor and Hill, 1989), and predictive validity (e.g., Horvath et al., 2011) of the measure. Cronbach’s alpha for the current sample was 0.89.

#### 2.2.4 Outcome Questionnaire-45.2 (OQ-45.2)

The OQ-45.2 (Lambert et al., 2004; see also Chiappelli et al., 2008) is a 45-item self-report instrument that was designed to measure important areas of functioning (i.e., symptoms, interpersonal problems, social role) of central interest to mental health. Each item is rated on a 5-point Likert scale ranging from 0 (*never*) to 4 (*almost always*). The sum of item scores (after reverse coding select items) provides a total score, which the present study used as the outcome index. Previous studies have demonstrated the good internal consistency and test–retest reliability of the measure (Doerfler et al., 2002). In the present study, Cronbach’s alpha for the OQ-45.2 total score was 0.87.

### 2.3 Residential treatment program

Patients were attending a multimodal residential treatment program with a main psychodynamic orientation applied to eating pathologies. Treatment included both individual and group psychotherapy (along with recreational and art therapy, social cooking, and social skills training), and the average treatment length was 175.9 days (*SD* = 67.31, range = 71–583). According to the most widespread practice guidelines for ED treatment, a team approach and patient-tailored perspective were the cornerstones of the therapeutic program (American Psychiatric Association, 2006). A multidisciplinary team of specialized professionals (i.e., psychiatrists, psychologists, social workers, nutritionists, physicians, and nurses) met on a weekly basis to discuss cases within a psychodynamic theoretical framework.

Each patient received individual psychotherapy once or twice a week on the basis of a comprehensive examination of their psychological development, psychodynamic issues, cognitive style, comorbid psychopathology, and family situation. Contents of the individual psychotherapy sessions included interventions that focused on patients’ feelings or desires, linked their current feelings and perceptions to past experiences in order to identify recurrent patterns, addressed patients’ use of symptoms to manage intense and painful emotions, and used the therapeutic relationship as both a source of information about patients’ interpersonal functioning and a vehicle through which to offer them a different model for relationships. In order to provide a therapeutic intervention that was tailored to each patient, therapists might also use more eclectic interventions such as psycho-education on ED symptoms, potential physical consequences of EDs, and treatment goals.

### 2.4 Procedures

During the first week of treatment, all patients were evaluated using the SCID-5-CV by a licensed staff psychiatrist and a clinical psychologist, to ensure fulfillment of the inclusion criteria. Patients were also asked to complete a self-report measure on psychological well-being. Additionally, height and weight were measured during a full medical examination, to calculate BMI. Moreover, at the same time point and during the week prior to discharge, all patients who agreed to participate completed self-report measures to assess overall psychopathological impairment. The therapeutic alliance was evaluated following the first four sessions of individual psychotherapy. Patients were asked to complete the self-report questionnaire on the therapeutic alliance referring to the clinician who was providing their individual psychotherapy. To reduce social desirability bias, patients were informed that their answers would not be shared with their treating clinicians, but would only be available to researchers. Participation was voluntary and written informed consent was provided by all study subjects prior to the assessments, following the review and approval of the study protocol by the local research ethics committee.

## 3 Statistical analyses

All analyses were performed using SPSS Version 27 for Windows and the *jAMM* package of the statistical software Jamovi (Gallucci, 2020), based on the R Studio package *lavaan* (Rosseel, 2019). Preliminarily, multivariate analyses of variance (MANOVAs) and bivariate correlations were conducted to check whether the PWB dimensions and early therapeutic alliance were influenced by the DSM-5 ED diagnosis (i.e., AN or BN), patient age, baseline BMI, previous hospitalizations for an ED, and treatment length. Subsequently, the clinical significance of therapeutic change was determined according to the criterion proposed by Jacobson and Truax (1991). To determine whether a patient’s change was reliable or the result of measurement error or chance, a reliable change index (RCI) was calculated by subtracting the post-treatment score from the pre-treatment score and dividing the resulting figure by the standard error of difference between the test scores. Patient change was considered reliable when it exceeded the measurement error at a 0.05 level of confidence. In the second step, a cut-off point (i.e., “criterion c”) was determined to assess whether a patient outcome score fell within the functional or dysfunctional population range. In the Italian population, the cut-off score was estimated as a total OQ-45.2 score of 66 (Chiappelli et al., 2008). Thus, the sample was classified into four outcome groups: (a) *clinically significant improvement*, with a pre-post decrease in the OQ-45.2 total score of more than 14 points and a cut-off score in the functional range; (b) *reliable improvement*, with a significant pre-post decrease in the OQ-45.2 total score but a cut-off score in the dysfunctional range; (c) *no change*, with no reliable change and a cut-off score in the dysfunctional range; and (d) *reliable deterioration*, with a pre-post increase in the OQ-45.2 total score of more than 14 points and a cut-off score in the dysfunctional range.

For the purpose of this study, and due to the small percentage of patients who deteriorated, patients were divided into three groups: those showing clinically significant change, those showing reliable therapeutic change, and those showing minimal change (i.e., unchanged and deteriorated patients).

Subsequently, multinomial logistic regression was used to investigate the direct effects of baseline PWB dimensions and the early therapeutic alliance on therapy outcome, with group membership at the end of therapy used as the outcome variable and scores for the six PWB dimensional scales and the WAI overall score used as predictors. The results were presented as odds ratios (ORs), showing how the likelihood of being in one outcome category changed when the value of the predictor was raised by one unit. If an OR was smaller than 1, the predictor was associated with a lower likelihood; in contrast, an OR greater than 1 suggested a higher likelihood by unit for the predictor.

Finally, separate mediation models of the relationship between pre-treatment PWB dimensions (i.e., PWB dimensional scale scores) and therapy outcome (i.e., OQ-45.2 total score) were tested to identify the mediating, indirect effect of the early therapeutic alliance (i.e., WAI overall score) on these relationships. A residualized change score was calculated for the OQ-45.2 total score by running a linear regression with the discharge values as the outcome and the baseline scores as the predictor. This score was then used in the mediation analyses. Bias-corrected 95% confidence intervals (CIs) were evaluated using the bootstrap percentiles method (*N* = 1,000), with effects considered significant when the resulting CI did not contain 0. All continuous variables were grand mean centered to reduce collinearity. Any patient lacking an OQ-45.2 assessment at treatment intake or discharge was excluded from the analyses.

## 4 Results

### 4.1 Preliminary analyses

A preliminary MANOVA revealed no significant differences between AN and BN patients in baseline PWB dimensions (Wilks’ *λ* [6,158] = 0.912, *p* = 0.22, η^2^_p_ = 0.04) and the early therapeutic alliance (Wilks’ *λ* [4,159] = 0.833, *p* = 0.51, η^2^*_p_* = 0.02). Similarly, no significant differences were found between those patients who were previously hospitalized in an ED unit and those who did not with respect to both baseline PWB dimensions (Wilks’ *λ* [6,158] = 1.504, *p* = 0.18, η^2^*_p_* = 0.05) and the early therapeutic alliance (Wilks’ λ [4,159] = 0.856, *p* = 0.49, η^2^p = 0.02). PWB dimensions were unrelated to baseline BMI (i.e., autonomy *r* = 0.06, *p* = 0.46; environmental mastery *r* = 0.02, *p* = 0.79; personal growth *r* = 0.03, *p* = 0.74; positive relations *r* = −0.03, *p* = 0.66; purpose in life *r* = 0.02, *p* = 0.82; self-acceptance *r* = −0.01, *p* = 0.92) and patient age (i.e., autonomy *r* = 0.14, *p* = 0.07; environmental mastery *r* = 0.08, *p* = 0.33; personal growth *r* = 0.05, *p* = 0.51; positive relations *r* = 0.10, *p* = 0.21; purpose in life *r* = 0.02, *p* = 0.85; self-acceptance *r* = 0.03, *p* = 0.74). Furthermore, no significant associations were found between baseline PWB dimensions and treatment length (i.e., autonomy *r* = −0.10, *p* = 0.18; environmental mastery *r* = −0.14, *p* = 0.09; personal growth *r* = −0.07, *p* = 0.39; purpose in life *r* = −0.11, *p* = 0.16; self-acceptance *r* = −0.12, *p* = 0.12), with the exception of the positive relations’ dimension (*r* = −0.19, *p* = 0.02). Lastly, the correlational analyses showed that the early therapeutic alliance was unrelated to patients’ baseline BMI (*r* = −0.08, *p* = 0.30) and age (*r* = 0.14, *p* = 0.07), as well as to treatment length (*r* = −0.13, *p* = 0.09).

### 4.2 Clinical significance of pre-post treatment symptomatic change

**Figure 1:**
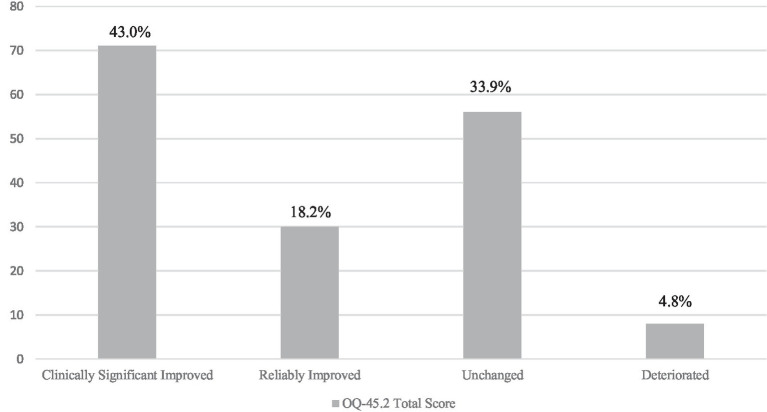
Clinical significance of change in overall symptomatic impairment (*N* = 165). Individual treatment response based on the Outcome Questionnaire-45.2 overall score (Lambert et al., 2004).

Figure 1 shows the results pertaining to the clinical significance of symptomatic change. In detail, 43% of patients (*n* = 71) met the twofold criterion for clinically significant improvement, and an additional 18.2% (*n* = 30) showed reliable symptomatic change, despite remaining within the dysfunctional range of functioning. On the other hand, 33.9% (*n* = 56) showed no significant change in overall symptomatic impairment and 6% (*n* = 8) deteriorated.

### 4.3 Direct effects of baseline psychological well-being and the early therapeutic alliance on therapy outcome

As described above, patients with no significant post-treatment change and those who deteriorated were grouped together. Table 1 shows the results of the multinomial logistic regression analyses predicting therapy outcome based on the clinical significance criterion. All variables were entered simultaneously. Of note, the early therapeutic alliance was strongly associated with a greater likelihood of clinically significant change (OR = 2.58, CI [0.33, 1.57]) and reliable change (OR = 2.80, CI [0.24, 1.82]), rather than no change and deterioration. PWB autonomy, positive relations, and self-acceptance were also connected to clinically significant change, rather than no change and deterioration (respectively OR = 1.04, CI [0.01, 0.07]; OR = 1.06, CI [0.01, 0.10]; and OR = 1.11, CI [0.02, 0.19]). On the other hand, PWB personal growth and self-acceptance were significantly associated with reliable change, rather than no change and deterioration (respectively OR = 1.07, CI [0.01, 1.22]; and OR = 1.06, CI [0.01, 0.11]). Moreover, PWB autonomy was associated with a lower likelihood of reliable change compared to no change and deterioration (OR = 0.95, CI [−0.09, −0.01]). No significant effects of PWB dimensions and the early therapeutic alliance emerged pertaining to the likelihood of clinically significant versus reliable change.

**Table 1:** Multinomial logistic regression analyses predicting individual treatment response (*N* = 165).

	Outcome groups		
	Clinically significant change vs. unchanged/deteriorated OR (95% CI)	Reliable change vs. unchanged/deteriorated OR (95% CI)	Clinically significant change vs. reliable change OR (95% CI)
*PWB*			
Autonomy	1.04 (0.002, 0.077)*	0.95 (−0.086, −0.006)*	1.01 (−0.024, 0.060)
Environmental mastery	1.01 (−0.042, 0.069)	0.98 (−0.076, 0.049)	1.02 (−0.043, 0.089)
Personal growth	1.03 (−0.018, 0.079)	1.07 (0.012, 0.122)**	0.96 (−0.099, 0.022)
Positive relations	1.06 (0.012, 0.099)**	0.98 (−0.064, 0.025)	0.98 (−0.076, 0.043)
Purpose in life	1.03 (−0.035, 0.090)	1.01 (−0.059, 0.077)	1.01 (−0.054, 0.092)
Self-acceptance	1.11 (0.022, 0.194)**	1.06 (0.008, 0.108)*	1.04 (−0.020, 0.099)
*WAI*			
Total score	2.58 (0.325, 1.572)***	2.80 (0.238, 1.823)**	1.01 (−0.949, 0.786)

* *p* ≤ 0.05, ** *p* ≤ 0.01, *** *p* ≤ 0.001.PWB, Psychological Well-Being scale (Ryff and Keyes, 1995); WAI, Working Alliance Inventory – Patient Version (WAI-P; Horvath and Greenberg, 1989). 95% CI, confidence interval; OR, odds ratio.The reference groups were unchanged/deteriorated patients and patients with reliable change.

### 4.4 Indirect effects of the early therapeutic alliance in the relationship between psychological well-being and therapy outcome

Table 2 displays the complete associations between the six PWB dimensions, the early therapeutic alliance, and therapy outcome (i.e., the residualized change score from the pre-post OQ-45.2 total score).

**Table 2:** Associations among psychological well-being dimensions, the therapeutic alliance, and therapy outcome (*N* = 165).

	1.		2.		3.		4.		5.		6.		7.		8.	
1.PWB autonomy	—														
2.PWB environmental mastery	0.534	***	—												
3.PWB personal growth	0.458	***	0.634	***	—										
4.PWB positive relations	0.493	***	0.665	***	0.581	***	—								
5.PWB purpose in life	0.447	***	0.845	***	0.717	***	0.613	***	—						
6.PWB self-acceptance	0.575	***	0.823	***	0.625	***	0.633	***	0.832	***	—				
7.WAI total score	0.231	**	0.284	***	0.360	***	0.391	***	0.341	***	0.308	***	—		
8.OQ-45.2 therapy outcome	−0.215	**	−0.434	***	−0.414	***	−0.357	***	−0.479	***	−0.485	***	−0.255	***	—

* *p* < 0.05, ** *p* < 0.01, *** *p* < 0.001.PWB, Psychological Well-Being scale (Ryff and Keyes, 1995); WAI, Working Alliance Inventory – Patient Version (WAI-P; Horvath and Greenberg, 1989); OQ-45.2, Outcome Questionnaire-45.2 (Lambert et al., 2004).

The six mediational models—one for each PWB dimension—revealed significant indirect effects of the therapeutic alliance in the relationships between different PWB components and therapy outcome (see Table 3). Additionally, higher levels of different PWB dimensions predicted a better therapy outcome through an increased therapeutic alliance. Specifically, the first mediation model showed that the point estimate of the indirect effect between PWB autonomy and therapy outcome through the early therapeutic alliance was −0.091 (*SE* = 0.04, *p* = 0.042), and the bootstrapped 95% CI [−0.196, −0.020] did not include 0, indicating that the indirect effect of the therapeutic alliance was significant. The total effect of the baseline PWB autonomy on therapy outcome was significant (*β* = −0.22, *p* = 0.006), whereas its direct effect was not significant after controlling for the mediator (*β* = −0.15, *p* = 0.077). Also in the second mediation model, greater PWB environmental mastery was associated with better therapy outcome, through a better therapeutic alliance (*β* = −0.07, *p* = 0.029). The total effect of the baseline PWB environmental mastery on therapy outcome was significant (*β* = −0.43, *p* < 0.001), as was its direct effect after controlling for the mediator (*β* = −0.35, *p* < 0.001). The indirect effect of PWB personal growth through the therapeutic alliance on therapy outcome was also significant (*β* = −0.09, *p* = 0.041). The total effect of the baseline PWB personal growth on therapy outcome was significant (*β* = −0.41, *p* < 0.001), as was its direct effect after controlling for the mediator (*β* = −0.31, *p* < 0.001). Similar findings emerged in the fourth mediation model, which found that the therapeutic alliance mediated the link between PWB positive relations and therapy outcome (*β* = −0.10, *p* = 0.025). Both the total and the direct effects of the baseline PW positive relations on therapy outcome were also significant (respectively *β* = −0.36, *p* < 0.001; and *β* = =0.30, *p* = 0.013). The indirect effect of PWB purpose in life through the therapeutic alliance on therapy outcome was also significant (*β* = −0.07, *p* = 0.042), as were the total and direct effects of PWB purpose in life on therapy outcome (respectively *β* = −0.48, *p* < 0.001; and *β* = −0.40, *p* < 0.001). Finally, greater PWB self-acceptance was associated with better therapy outcome, through a better therapeutic alliance (*β* = −0.07, *p* = 0.020). The total effect of the baseline PWB self-acceptance on therapy outcome was significant (*β* = −0.49, *p* < 0.001), as was its direct effect after controlling for the mediator (*β* = −0.42, *p* < 0.001).

**Table 3:** Mediation of the early therapeutic alliance in the relationship between psychological well-being dimensions and therapy outcome (*N* = 165).

Type	Effect	Estimate	*SE*	95% CI		*β*	*p*
				Lower	Upper		
Indirect	Autonomy ⇒ alliance ⇒ outcome	−0.091	0.043	−0.196	−0.020	−0.059	0.042
Component	Autonomy ⇒ alliance	0.012	0.005	0.002	0.022	0.192	0.004
	Alliance ⇒ outcome	−7.437	2.180	−12.317	−3.762	−0.309	< 0.001
Direct	Autonomy ⇒ outcome	−0.231	0.130	−0.478	−0.016	−0.151	0.077
Total	Autonomy ⇒ outcome	−0.327	0.118	−0.532	−0.082	−0.215	0.006
Indirect	Environmental mastery ⇒ alliance ⇒ outcome	−0.106	0.048	−0.216	−0.021	−0.074	0.029
Component	Environmental mastery ⇒ alliance	0.020	0.005	0.011	0.031	0.341	<0.001
	Alliance ⇒ outcome	−5.214	2.309	−10.196	−1.141	−0.216	0.024
Direct	Environmental mastery ⇒ outcome	−0.513	0.128	−0.766	−0.258	−0.355	<0.001
Total	Environmental mastery ⇒ outcome	−0.622	0.106	−0.838	−0.409	−0.434	<0.001
Indirect	Personal Growth ⇒ Alliance ⇒ Outcome	−0.155	0.077	−0.329	−0.024	−0.086	0.041
Component	Personal Growth ⇒ Alliance	0.031	0.007	0.019	0.046	0.422	<0.001
	Alliance ⇒ Outcome	−4.911	2.171	−9.921	−0.869	−0.204	0.024
Direct	Personal Growth ⇒ Outcome	−0.571	0.148	−0.874	−0.287	−0.317	<0.001
Total	Personal Growth ⇒ Outcome	−0.735	0.119	−0.966	−0.515	−0.414	<0.001
Indirect	Positive Relations ⇒ Alliance ⇒ Outcome	−0.151	0.068	−0.291	−0.024	−0.095	0.025
Component	Positive Relations ⇒ Alliance	0.027	0.005	0.018	0.037	0.413	<0.001
	Alliance ⇒ outcome	−5.555	2.589	−11.055	−0.910	−0.230	0.032
Direct	Positive relations ⇒ outcome	−0.413	0.165	−0.751	−0.105	−0.296	0.013
Total	Positive relations ⇒ outcome	−0.570	0.133	−0.832	−0.319	−0.356	<0.001
Indirect	Purpose in life ⇒ alliance ⇒ outcome	−0.106	0.053	−0.224	−0.018	−0.069	0.042
Component	Purpose in life ⇒ alliance	0.023	0.005	0.014	0.035	0.360	<0.001
	Alliance ⇒ outcome	−4.635	2.285	−9.667	−0.840	−0.192	0.043
Direct	Purpose in life ⇒ outcome	−0.618	0.136	−0.887	−0.355	−0.403	<0.001
Total	Purpose in life ⇒ outcome	−0.724	0.108	−0.931	−0.508	−0.479	<0.001
Indirect	Self-acceptance ⇒ alliance ⇒ outcome	−0.085	0.036	−0.155	−0.013	−0.065	0.020
Component	Self-acceptance ⇒ alliance	0.017	0.004	0.009	0.025	0.322	<0.001
	Alliance ⇒ outcome	−4.890	1.749	−8.318	−1.462	−0.203	0.005
Direct	Self-acceptance ⇒ outcome	−0.544	0.094	−0.728	−0.360	−0.419	<0.001
Total	Self-acceptance ⇒ outcome	−0.627	0.090	−0.804	−0.450	−0.485	<0.001

CI, confidence interval; SE, standardized error.Confidence intervals computed using bootstrap percentiles.

## 5 Discussion

To the best of our knowledge, the present study was the first to investigate both the role of baseline PWB dimensions in determining individual responses to ED treatment and the indirect effects of the early therapeutic alliance in the relationship between the theoretically grounded concept of eudaimonic psychological well-being and ED therapy outcomes. Although previous studies have included measures of subjective well-being and health-related dimensions (e.g., Jenkins et al., 2011; Mond et al., 2012), or compared PWB dimensions in ED patients to those of healthy controls (e.g., Tomba et al., 2014), the present research shed new light on the relationship between ED patients’ dimensions of positive psychological functioning and their trusting, collaborative relationships with treating clinicians in determining symptomatic change through psychotherapy. The findings align with broader recognition that outcome research in the field of EDs is dramatically biased towards psychopathological dysfunction, on the assumption that mental health is predicated on the absence of illness rather than the presence of psychological wellness (Fava and Tomba, 2009; de Vos et al., 2018). Additionally, evidence relating the therapeutic alliance to ED treatment outcome are of limited clinical use without a better understanding of the facilitators or barriers to therapeutic alliance formation in ED patients (Lev Ari et al., 2024).

Applying the clinical significance criterion of therapeutic change (Jacobson and Truax, 1991), the present study found that 43% of patients experienced clinically significant symptomatic improvement (shifting into the functional population range) and a further 18.2% responded reliably in terms of overall symptom reduction. However, a significant percentage of patients (33.9%) remained unchanged and an additional 4.8% showed reliable deterioration. These findings are congruent with previous studies on ED-specific residential treatment settings (Muzi et al., 2020) and inpatient facilities (Schlegl et al., 2014, 2016). Of note, when exploring the direct effects of PWB dimensions on individual treatment response, the results showed that patients’ capacity to be self-determined and adhere to personal standards, to establish empathic, warm, and intimate relationships with others, and to maintain a nuanced and compassionate attitude towards the self were related to clinically significant change. In contrast, self-acceptance and personal growth (i.e., openness to new experiences and the realization of personal potential) were associated with reliable change. Previous ED research has shown that patients with greater identity confusion, greater difficulty distinguishing internal representations from external circumstances, more severe impairment in interpersonal functioning, and lower self-compassion are more likely to experience poor therapeutic outcomes (Kelly et al., 2013; Verschueren et al., 2017; Muzi et al., 2021). On the other hand, a higher baseline ability to construct a coherent and meaningful personal narrative, better interpersonal functioning, and a sense of directedness and purpose has been shown to be related to better therapeutic outcomes (Vall and Wade, 2015; Marco et al., 2019; Muzi et al., 2021). Thus, interventions targeting these areas of psychological strength may have pivotal clinical implications, increasing motivation, treatment adherence, and therapeutic progress.

The present findings also showed that the early therapeutic alliance (i.e., within the first four therapy sessions) was associated with clinically significant and reliable change. Despite substantial evidence highlighting a potential alliance-outcome association in EDs (Zaitsoff et al., 2015; Graves et al., 2017), the precise implications of this association remain unclear. A possible explanation for the present result may lie in the time point at which this variable was measured: as the therapeutic alliance begins to form from the first meeting between therapist and patient, and early phases of the therapeutic relationship have been found to predict positive psychotherapy outcomes later on (Werz et al., 2022), the early therapeutic alliance may be significant to the relationship between the therapeutic alliance and therapy outcome over time. Furthermore, this direct effect may be particularly relevant to specialized, full-time residential ED treatment settings, which are more likely to reward strong interpersonal relationships with members of the treatment team and other patients, compared to less intensive care settings (e.g., outpatient treatments, day treatment programs) (Peckmezian and Paxton, 2020).

With respect to the second aim of the study, the early therapeutic alliance also emerged as a significant mediator in the relationship between all baseline PWB dimensions and overall symptomatic change. While some studies have suggested an association between the therapeutic alliance and Ryff’s concept of psychological well-being (Prusiński, 2022), evidence beyond the mental health context also suggests that the doctor–patient alliance, defined by agreement over treatment goals and tasks and mutual trust and liking, may be associated with patients’ life satisfaction and quality of life (Fuertes et al., 2009). Additionally, the present results align with evidence showing the indirect effect of the therapeutic alliance on therapeutic change, despite heterogeneity between study designs, clinical populations, and treatment settings (Baier et al., 2020). These findings may have significant clinical implications for ED treatment. First, patients’ baseline ability to build deep and trust-based relations with others may significantly impact their contribution to the collaborative relationships with their treating clinicians over the course of therapy, then, in turn, leading to better outcomes (Hersoug et al., 2013). Moreover, a sense of agency and the capacity to cope with complex environmental factors may influence, through a better therapeutic alliance, the therapeutic dyad’s ability to tailor the therapeutic space to support the intense work process needed to recover from EDs (Bardone-Cone et al., 2018). This is also consistent with Ryff’s (1989) psychological well-being model, which outlines that the most crucial factor for human development is an increase in the sense of self-directedness and the ability to transform the environment in accordance with one’s values and needs. Furthermore, a positive but realistic attitude towards oneself and effective intrinsic behavioral regulation may help to foster the therapeutic relationship as an important stabilizing condition to counteract ED patients’ fluctuating levels of subjective discomfort and ambivalence towards change (Lev Ari et al., 2024).

Notwithstanding these promising results, the present study suffered from several limitations that must be acknowledged in the interpretation of the results. First, all patients were recruited from a single residential ED treatment program, which hinders the generalizability of the findings to patients in other ED treatment settings (e.g., day programs, outpatient settings) and individuals suffering from other baseline EDs other than AN and BN (e.g., binge eating disorder, avoidant/restrictive food intake disorder). Furthermore, despite patients were asked to evaluate the perceived therapeutic alliance referring to clinicians who were providing individual psychotherapy, the multidisciplinary nature of the overall therapeutic approach may still represent a potential confounding variable (Elran-Barak et al., 2024). Second, the study only included White assigned females at birth, which limits the generalizability of the findings to assigned males, minority demographic populations, and individuals with other gender identities (e.g., Mirabella et al., 2024). Third, the present study only relied on self-report measures, which may be susceptible to response bias. Therefore, future research should address the study hypotheses using more diverse samples with different ED diagnoses using multi-informant assessment methods, while also controlling for the treatment setting and therapeutic approach (e.g., psychodynamic, cognitive-behavioral, interpersonal). Given increasing evidence of relevant clinical challenges with less-studied ED diagnoses (Kambanis and Thomas, 2023; Solmi et al., 2024), further replication considering other ED patient groups is especially needed. Fourth, future research should also include therapists’ and/or external ratings of the early therapeutic alliance. As the therapeutic alliance reflects a dyadic process in which both the patient and the therapist continuously perceive and influence each other’s perspectives (Atzil-Slonim et al., 2015), it would be crucial to also examine the level of congruence between patient and therapist perspectives on the therapeutic alliance within ED treatment (e.g., Whelen et al., 2022). Of note, due to the complex nature of EDs, measures of therapeutic change should also take into account pre-post data on other parameters such as BMI and number of dietary restrictions, inappropriate compensatory behaviors or binge eating episodes per week. Future studies should also investigate the potential role of other relevant baseline variables (e.g., duration of illness; Broomfield et al., 2017), as well as the influence of individual factors such as attachment patterns (e.g., Tasca, 2019) or early traumatic experiences (e.g., Musetti et al., 2023), on both PWB dimensions and early therapeutic alliance.

Despite these limitations, the present study, which applied a theoretically grounded model of psychological well-being, enhances our understanding of the complex interplay between ED patients’ positive psychological functioning, the early therapeutic alliance, and therapeutic change at both individual and overall levels. From a clinical standpoint, interventions targeting and promoting experiences of well-being may address the growing awareness that standard, symptom-oriented treatment for EDs may not be sufficient to determine a full recovery (Fava, 2016; Bardone-Cone et al., 2018). Conversely, previous studies have shown promising results on the effectiveness of interventions aimed at improving eudaemonic well-being in both clinical and nonclinical populations (van Dierendonck and Lam, 2023). Since EDs still represent a major global clinical and public health challenge, these findings suggest that current therapeutic programs might benefit from including interventions aimed at increasing overall positive human functioning. Such observations seem particularly relevant for residential treatments, which are commonly administered to patients with chronic, severe, and enduring forms of eating pathologies that are more likely to be unresponsive to treatment approaches that are solely symptom-oriented. Furthermore, a stronger alliance in the earliest stages of treatment appears to be an independent and interactive predictor of symptom reduction and one of the many processes driving change in ED patients. By identifying patient factors that may positively or negatively influence the therapeutic alliance in ED treatment, therapists may better prevent relationship ruptures and tailor their interventions to overcome these (Safran et al., 2011; Lingiardi et al., 2018; Colli et al., 2019), thereby enhancing therapeutic outcomes for this difficult-to-treat population.

## Data availability statement

The raw data supporting the conclusions of this article will be made available by the authors, without undue reservation.

## Ethics statement

The studies involving humans were approved by Research Ethics Committee of the Department of Dynamic and Clinical Psychology, and Health Studies, Sapienza University of Rome; reference number: 0000398. The studies were conducted in accordance with the local legislation and institutional requirements. The participants provided their written informed consent to participate in this study.

## Author contributions

LM: Conceptualization, Methodology, Writing – original draft, Writing – review & editing. NC: Data curation, Formal analysis, Methodology, Software, Writing – review & editing. MM: Investigation, Methodology, Supervision, Writing – review & editing. AF: Investigation, Project administration, Supervision, Writing – review & editing. MR: Methodology, Project administration, Supervision, Writing – review & editing. CM: Investigation, Methodology, Supervision, Writing – review & editing. VL: Conceptualization, Methodology, Project administration, Supervision, Writing – review & editing.

## References

[ref1] Ágh T. Kovács G. Supina D. Pawaskar M. Herman B. K. Vokó Z.. (2016). A systematic review of the health-related quality of life and economic burdens of anorexia nervosa, bulimia nervosa, and binge eating disorder. Eat. Weight Disord. 21, 353–364. doi: 10.1007/s40519-016-0264-x, PMID: 26942768 PMC5010619

[ref2] American Psychiatric Association (2006). Treatment of patients with eating disorders, third edition. American Psychiatric Association. Am. J. Psychiatry 163, 4–54, 16925191

[ref3] Antoniou P. Cooper M. (2013). Psychological treatments for eating disorders: what is the importance of the quality of the therapeutic alliance for outcomes. Couns. Psychol. Rev. 28, 34–46. doi: 10.53841/bpscpr.2013.28.4.34

[ref4] Atzil-Slonim D. Bar-Kalifa E. Rafaeli E. Lutz W. Rubel J. Schiefele A. K.. (2015). Therapeutic bond judgments: congruence and incongruence. J. Consult. Clin. Psychol. 83, 773–784. doi: 10.1037/ccp0000015 25664641

[ref5] Baier A. L. Kline A. C. Feeny N. C. (2020). Therapeutic alliance as a mediator of change: a systematic review and evaluation of research. Clin. Psychol. Rev. 82:101921. doi: 10.1016/j.cpr.2020.101921, PMID: 33069096

[ref6] Bardone-Cone A. M. Hunt R. A. Watson H. J. (2018). An overview of conceptualizations of eating disorder recovery, recent findings, and future directions. Curr. Psychiatry Rep. 20:79. doi: 10.1007/s11920-018-0932-9, PMID: 30094740

[ref7] Blasco-Belled A. Alsinet C. (2022). The architecture of psychological well-being: a network analysis study of the Ryff psychological well-being scale. Scand. J. Psychol. 63, 199–207. doi: 10.1111/sjop.12795, PMID: 35041208

[ref8] Bordin E. S. (1979). The generalizability of the psychoanalytic concept of the working alliance. Psychother. Theory Res. Pract. 16, 252–260. doi: 10.1037/h0085885

[ref9] Broomfield C. Stedal K. Touyz S. Rhodes P. (2017). Labeling and defining severe and enduring anorexia nervosa: a systematic review and critical analysis. Int. J. Eat. Disord. 50, 611–623. doi: 10.1002/eat.22715, PMID: 28444828

[ref10] Brown A. Mountford V. Waller G. (2013). Therapeutic alliance and weight gain during cognitive behavioural therapy for anorexia nervosa. Behav. Res. Ther. 51, 216–220. doi: 10.1016/j.brat.2013.01.008, PMID: 23435122

[ref11] Browne J. Penn D. L. Meyer-Kalos P. S. Mueser K. T. Estroff S. E. Brunette M. F.. (2017). Psychological well-being and mental health recovery in the NIMH RAISE early treatment program. Schizophr. Res. 185, 167–172. doi: 10.1016/j.schres.2016.11.032, PMID: 27913160 PMC5612365

[ref12] Chiappelli M. Lo Coco G. Gullo S. Bensi L. Prestano C. (2008). The outcome questionnaire 45.2. Italian validation of an instrument for the assessment of psychological treatments. Epidemiol. Psichiatr. Soc. 17, 152–161. doi: 10.1017/S1121189X00002852, PMID: 18589632

[ref13] Colli A. Gentile D. Condino V. Lingiardi V. (2019). Assessing alliance ruptures and resolutions: reliability and validity of the collaborative interactions scale-revised version. Psychother. Res. 29, 279–292. doi: 10.1080/10503307.2017.1414331, PMID: 29271303

[ref14] Colli A. Speranza A. M. Lingiardi V. Gentile D. Nassisi V. Hilsenroth M. J. (2015). Eating disorders and therapist emotional responses. J. Nerv. Ment. Dis. 203, 843–849. doi: 10.1097/NMD.0000000000000379, PMID: 26461481

[ref15] de la Rie S. M. Noordenbos G. van Furth E. F. (2005). Quality of life and eating disorders. Qual. Life Res. 14, 1511–1521. doi: 10.1007/s11136-005-0585-0 16110931

[ref16] de Vos J. A. Radstaak M. Bohlmeijer E. T. Westerhof G. J. (2018). Having an eating disorder and still being able to flourish? Examination of pathological symptoms and well-being as two continua of mental health in a clinical sample. Front. Psychol. 9:2145. doi: 10.3389/fpsyg.2018.02145, PMID: 30498463 PMC6249270

[ref17] Del Re A. C. Flückiger C. Horvath A. O. Wampold B. E. (2021). Examining therapist effects in the alliance-outcome relationship: a multilevel meta-analysis. J. Consult. Clin. Psychol. 89, 371–378. doi: 10.1037/ccp0000637, PMID: 33829817

[ref18] Doerfler L. A. Addis M. E. Moran P. W. (2002). Evaluating mental health outcomes in an inpatient setting: convergent and divergent validity of the OQ-45 and BASIS-32. J. Behav. Health Serv. Res. 29, 394–403. doi: 10.1007/BF02287346, PMID: 12404934

[ref19] Doll H. A. Petersen S. E. Stewart-Brown S. L. (2005). Eating disorders and emotional and physical well-being: associations between student self-reports of eating disorders and quality of life as measured by the SF-36. Qual. Life Res. 14, 705–717. doi: 10.1007/s11136-004-0792-0, PMID: 16022064

[ref20] Elran-Barak R. Grundman-Shem Tov R. Zubery E. Lewis Y. D. (2024). Therapeutic alliance with psychotherapist versus dietician: a pilot study of eating disorder treatment in a multidisciplinary team during the COVID-19 pandemic. Front. Psych. 14:1267676. doi: 10.3389/fpsyt.2023.1267676, PMID: 38348361 PMC10860050

[ref21] Fava G. A. (2016). Well-being therapy: current indications and emerging perspectives. Psychother. Psychosom. 85, 136–145. doi: 10.1159/000444114 27043240

[ref22] Fava G. A. Tomba E. (2009). Increasing psychological well-being and resilience by psychotherapeutic methods. J. Pers. 77, 1903–1934. doi: 10.1111/j.1467-6494.2009.00604.x, PMID: 19807860

[ref23] First M. B. Williams J. B. W. Karg R. S. Spitzer R. L. (2016). User’s guide for the SCID-5-CV structured clinical interview for DSM-5® disorders: Clinical version. Arlington, VA: American Psychiatric Publishing.

[ref24] Flückiger C. Del Re A. C. Wampold B. E. Horvath A. O. (2018). The alliance in adult psychotherapy: a meta-analytic synthesis. Psychotherapy 55, 316–340. doi: 10.1037/pst0000172, PMID: 29792475

[ref25] Fossati A. Borroni S. (2017). SCID-5-CV. Intervista clinica strutturata per i disturbi del DSM-5®. Versione per il clinico. Milan: Raffaello Cortina.

[ref26] Fuertes J. N. Boylan L. S. Fontanella J. A. (2009). Behavioral indices in medical care outcome: the working alliance, adherence, and related factors. J. Gen. Intern. Med. 24, 80–85. doi: 10.1007/s11606-008-0841-4, PMID: 18972089 PMC2607493

[ref27] Gallucci M. (2020). JAMM: jamovi advanced mediation models. [jamovi module]. Available at: https://jamovi-amm.github.io/

[ref28] Graves T. A. Tabri N. Thompson-Brenner H. Franko D. L. Eddy K. T. Bourion-Bedes S.. (2017). A meta-analysis of the relation between therapeutic alliance and treatment outcome in eating disorders. Int. J. Eat. Disord. 50, 323–340. doi: 10.1002/eat.22672, PMID: 28152196

[ref29] Groth T. Hilsenroth M. J. Gold J. Boccio D. Tasca G. A. (2020). Therapist factors related to the treatment of adolescent eating disorders. Prof. Psychol. Res. Pract. 51, 517–526. doi: 10.1037/pro0000308

[ref30] Hanson W. E. Curry K. T. Bandalos D. L. (2002). Reliability generalization of working Alliance inventory scale scores. Educ. Psychol. Meas. 62, 659–673. doi: 10.1177/0013164402062004008

[ref31] Hersoug A. G. Høglend P. Gabbard G. O. Lorentzen S. (2013). The combined predictive effect of patient characteristics and alliance on long-term dynamic and interpersonal functioning after dynamic psychotherapy. Clin. Psychol. Psychother. 20, 297–307. doi: 10.1002/cpp.1770, PMID: 22298434

[ref32] Hilbert A. Hoek H. W. Schmidt R. (2017). Evidence-based clinical guidelines for eating disorders: international comparison. Curr. Opin. Psychiatry 30, 423–437. doi: 10.1097/YCO.0000000000000360, PMID: 28777107 PMC5690314

[ref33] Horvath A. O. Del Re A. C. Flückiger C. Symonds D. (2011). Alliance in individual psychotherapy. Psychotherapy 48, 9–16. doi: 10.1037/a0022186 21401269

[ref34] Horvath A. O. Greenberg L. S. (1989). Development and validation of the working Alliance inventory. J. Couns. Psychol. 36, 223–233. doi: 10.1037/0022-0167.36.2.223

[ref35] Jacobson N. S. Truax P. (1991). Clinical significance: a statistical approach to defining meaningful change in psychotherapy research. J. Consult. Clin. Psychol. 59, 12–19. doi: 10.1037//0022-006x.59.1.12, PMID: 2002127

[ref36] Jenkins P. E. Hoste R. R. Meyer C. Blissett J. M. (2011). Eating disorders and quality of life: a review of the literature. Clin. Psychol. Rev. 31, 113–121. doi: 10.1016/j.cpr.2010.08.003 20817335

[ref37] Jones A. Lindekilde N. Lübeck M. Clausen L. (2015). The association between interpersonal problems and treatment outcome in the eating disorders: a systematic review. Nord. J. Psychiatry 69, 563–573. doi: 10.3109/08039488.2015.1019924, PMID: 25768663

[ref38] Kambanis P. E. Thomas J. J. (2023). Assessment and treatment of avoidant/restrictive food intake disorder. Curr. Psychiatry Rep. 25, 53–64. doi: 10.1007/s11920-022-01404-6, PMID: 36640211 PMC10211369

[ref39] Katznelson H. Falkenström F. Daniel S. Lunn S. Folke S. Pedersen S. H.. (2020). Reflective functioning, psychotherapeutic alliance, and outcome in two psychotherapies for bulimia nervosa. Psychotherapy 57, 129–140. doi: 10.1037/pst0000245, PMID: 31599637

[ref40] Kelly A. C. Carter J. C. Zuroff D. C. Borairi S. (2013). Self-compassion and fear of self-compassion interact to predict response to eating disorders treatment: a preliminary investigation. Psychother. Res. 23, 252–264. doi: 10.1080/10503307.2012.717310, PMID: 22917037

[ref41] Lambert M. J. Gregersen A. T. Burlingame G. M. (2004). “The Outcome Questionnaire-45” in The use of psychological testing for treatment planning and outcomes assessment: Instruments for adults. ed. Maruish M. E.. 3rd ed (Mahwah, NJ: Lawrence Erlbaum Associates), 191–234.

[ref42] Lev Ari H. S. Gur E. Lotan-Wohl M. Bernstein Z. L. Tuval-Mashiach R. (2024). Facilitators and obstacles to therapeutic alliance among patients with anorexia nervosa: a qualitative dyadic research. Couns. Psychother. Res. 24, 136–153. doi: 10.1002/capr.12617

[ref43] Lingiardi V. (2002). L’alleanza terapeutica: Teoria, clinica, ricerca. 1st Edn. Milan: Raffaello Cortina.

[ref44] Lingiardi V. Colli A. Muzi L. (2018). A clinically useful assessment of patients’ (and therapists’) mental functioning: M-axis implications for the therapeutic alliance. Psychoanal. Psychol. 35, 306–314. doi: 10.1037/pap0000200

[ref45] Lingiardi V. McWilliams N. (2017). Psychodynamic diagnostic manual: PDM-2. 2nd Edn. New York, NY: Guilford Press.

[ref46] Marco J. H. Cañabate M. Pérez S. (2019). Meaning in life is associated with the psychopathology of eating disorders: differences depending on the diagnosis. Eat. Disord. 27, 550–564. doi: 10.1080/10640266.2018.1560852, PMID: 30663525

[ref47] Marzola E. Albini E. Delsedime N. Fassino S. Abbate-Daga G. (2019). Therapeutic alliance in inpatients with severe anorexia nervosa. Eur. Eat. Disord. Rev. 27, 671–681. doi: 10.1002/erv.2687, PMID: 31172605

[ref48] Mason T. B. Dayag R. Dolgon-Krutolow A. Lam K. Zhang D. (2022). A systematic review of maladaptive interpersonal behaviors and eating disorder psychopathology. Eat. Behav. 45:101601. doi: 10.1016/j.eatbeh.2022.101601, PMID: 35221209

[ref49] Mirabella M. Carone N. Franco A. Rugo M. A. Speranza A. M. Mazzeschi C.. (2024). Emotional dysregulation and eating symptoms in gender dysphoria and eating disorders: the mediating role of body uneasiness. Curr. Psychol. 43, 17090–17104. doi: 10.1007/s12144-024-05663-9

[ref50] Mirabella M. Muzi L. Franco A. Urgese A. Rugo M. A. Mazzeschi C.. (2023). From symptoms to subjective and bodily experiences: the contribution of the psychodynamic diagnostic manual (PDM-2) to diagnosis and treatment monitoring in eating disorders. Eat. Weight Disord. 28:35. doi: 10.1007/s40519-023-01562-3, PMID: 36997702 PMC10063489

[ref51] Mond J. Hay P. Rodgers B. Owen C. (2012). Quality of life impairment in a community sample of women with eating disorders. Aust. N. Z. J. Psychiatry 46, 561–568. doi: 10.1177/0004867411433967, PMID: 22679207

[ref52] Musetti A. Gagliardini G. Lenzo V. Cella S. (2023). From childhood emotional maltreatment to disordered eating: a path analysis. Psychoanal. Psychol. 40, 90–98. doi: 10.1037/pap0000438

[ref53] Muzi L. Tieghi L. Rugo M. A. Lingiardi V. (2020). Evaluating empirically valid and clinically meaningful change in intensive residential treatment for severe eating disorders at discharge and at a 6-month follow-up. Eat. Weight Disord. 25, 1609–1620. doi: 10.1007/s40519-019-00798-2 31673985

[ref54] Muzi L. Tieghi L. Rugo M. Lingiardi V. (2021). Eating pathology and psychodynamic diagnostic manual (PDM-2) diagnostic assessment: implications for treatment outcomes in a psychodynamic-oriented residential setting. Psychoanal. Psychol. 38, 300–310. doi: 10.1037/pap0000337

[ref55] Osório F. L. Loureiro S. R. Hallak J. E. C. Machado-de-Sousa J. P. Ushirohira J. M. Baes C. V. W.. (2019). Clinical validity and intrarater and test-retest reliability of the structured clinical interview for DSM-5 – clinician version (SCID-5-CV). Psychiatry Clin. Neurosci. 73, 754–760. doi: 10.1111/pcn.12931, PMID: 31490607

[ref56] Peckmezian T. Paxton S. J. (2020). A systematic review of outcomes following residential treatment for eating disorders. Eur. Eat. Disord. Rev. 28, 246–259. doi: 10.1002/erv.2733, PMID: 32196843 PMC7216912

[ref57] Prusiński T. (2022). The strength of alliance in individual psychotherapy and patient’s wellbeing: the relationships of the therapeutic alliance to psychological wellbeing, satisfaction with life, and flourishing in adult patients attending individual psychotherapy. Front. Psych. 13:827321. doi: 10.3389/fpsyt.2022.827321, PMID: 35173642 PMC8842997

[ref58] Raykos B. C. McEvoy P. M. Erceg-Hurn D. Byrne S. M. Fursland A. Nathan P. (2014). Therapeutic alliance in enhanced cognitive behavioural therapy for bulimia nervosa: probably necessary but definitely insufficient. Behav. Res. Ther. 57, 65–71. doi: 10.1016/j.brat.2014.04.004, PMID: 24841726

[ref59] Rosseel Y. (2019). Lavaan: an R package for structural equation modeling. J. Stat. Softw. 48, 1–36. doi: 10.18637/jss.v048.i02

[ref60] Ruini C. Ottolini F. Rafanelli C. Tossani E. Ryff C. D. Fava G. A. (2003). The relationship of psychological well-being to distress and personality. Psychother. Psychosom. 72, 268–275. doi: 10.1159/000071898 12920331

[ref61] Ryan R. M. Deci E. L. (2001). On happiness and human potentials: a review of research on hedonic and eudaimonic well-being. Annu. Rev. Psychol. 52, 141–166. doi: 10.1146/annurev.psych.52.1.141, PMID: 11148302

[ref62] Ryff C. D. (1989). Happiness is everything, or is it? Explorations on the meaning of psychological well-being. J. Pers. Soc. Psychol. 57, 1069–1081. doi: 10.1037/0022-3514.57.6.1069

[ref63] Ryff C. D. (2014). Psychological well-being revisited: advances in the science and practice of eudaimonia. Psychother. Psychosom. 83, 10–28. doi: 10.1159/000353263, PMID: 24281296 PMC4241300

[ref64] Ryff C. D. Keyes C. L. (1995). The structure of psychological well-being revisited. J. Pers. Soc. Psychol. 69, 719–727. doi: 10.1037//0022-3514.69.4.719 7473027

[ref65] Ryff C. D. Singer B. (1996). Psychological well-being: meaning, measurement, and implications for psychotherapy research. Psychother. Psychosom. 65, 14–23. doi: 10.1159/000289026, PMID: 8838692

[ref66] Ryff C. D. Singer B. H. (2006). Best news yet on the six-factor model of well-being. Soc. Sci. Res. 35, 1103–1119. doi: 10.1016/j.ssresearch.2006.01.002

[ref67] Safran J. D. Muran J. C. Eubanks-Carter C. (2011). Repairing alliance ruptures. Psychotherapy 48, 80–87. doi: 10.1037/a0022140 21401278

[ref68] Schlegl S. Diedrich A. Neumayr C. Fumi M. Naab S. Voderholzer U. (2016). Inpatient treatment for adolescents with anorexia nervosa: clinical significance and predictors of treatment outcome. Eur. Eat. Disord. Rev. 24, 214–222. doi: 10.1002/erv.2416, PMID: 26603278

[ref69] Schlegl S. Quadflieg N. Löwe B. Cuntz U. Voderholzer U. (2014). Specialized inpatient treatment of adult anorexia nervosa: effectiveness and clinical significance of changes. BMC Psychiatry 14:258. doi: 10.1186/s12888-014-0258-z, PMID: 25193513 PMC4172844

[ref70] Solmi M. Monaco F. Højlund M. Monteleone A. M. Trott M. Firth J.. (2024). Outcomes in people with eating disorders: a transdiagnostic and disorder-specific systematic review, meta-analysis and multivariable meta-regression analysis. World Psychiatry 23, 124–138. doi: 10.1002/wps.21182, PMID: 38214616 PMC10785991

[ref71] Tasca G. A. (2019). Attachment and eating disorders: a research update. Curr. Opin. Psychol. 25, 59–64. doi: 10.1016/j.copsyc.2018.03.003, PMID: 29579723

[ref72] Tichenor V. Hill C. E. (1989). A comparison of six measures of working alliance. Psychother. Theory Res. Pract. Train. 26, 195–199. doi: 10.1037/h0085419

[ref73] Tomba E. Offidani E. Tecuta L. Schumann R. Ballardini D. (2014). Psychological well-being in out-patients with eating disorders: a controlled study. Int. J. Eat. Disord. 47, 252–258. doi: 10.1002/eat.22197, PMID: 24123214

[ref74] Tomba E. Tecuta L. Schumann R. Ballardini D. (2017). Does psychological well-being change following treatment? An exploratory study on outpatients with eating disorders. Compr. Psychiatry 74, 61–69. doi: 10.1016/j.comppsych.2017.01.001, PMID: 28107643

[ref75] Treasure J. Hübel C. Himmerich H. (2022). The evolving epidemiology and differential etiopathogenesis of eating disorders: implications for prevention and treatment. World Psychiatry 21, 147–148. doi: 10.1002/wps.20935, PMID: 35015360 PMC8751578

[ref76] Vaillant G. E. (2012). Positive mental health: is there a cross-cultural definition? World Psychiatry 11, 93–99. doi: 10.1016/j.wpsyc.2012.05.006, PMID: 22654934 PMC3363378

[ref77] Vall E. Wade T. D. (2015). Predictors of treatment outcome in individuals with eating disorders: a systematic review and meta-analysis. Int. J. Eat. Disord. 48, 946–971. doi: 10.1002/eat.22411, PMID: 26171853

[ref78] van Dierendonck D. Lam H. (2023). Interventions to enhance eudaemonic psychological well-being: a meta-analytic review with Ryff's scales of psychological well-being. Appl. Psychol. Health Well Being 15, 594–610. doi: 10.1111/aphw.12398, PMID: 36073601

[ref79] Verschueren M. Luyckx K. Kaufman E. A. Vansteenkiste M. Moons P. Sleuwaegen E.. (2017). Identity processes and statuses in patients with and without eating disorders. Eur. Eat. Disord. Rev. 25, 26–35. doi: 10.1002/erv.2487, PMID: 27790863

[ref80] Waller G. Evans J. Stringer H. (2012). The therapeutic alliance in the early part of cognitive-behavioral therapy for the eating disorders. Int. J. Eat. Disord. 45, 63–69. doi: 10.1002/eat.20914, PMID: 21321989

[ref81] Werz J. Voderholzer U. Tuschen-Caffier B. (2022). Alliance matters: but how much? A systematic review on therapeutic alliance and outcome in patients with anorexia nervosa and bulimia nervosa. Eat. Weight Disord. 27, 1279–1295. doi: 10.1007/s40519-021-01281-7, PMID: 34374966 PMC9079014

[ref82] Whelen M. L. Ezawa I. D. Strunk D. R. (2022). Congruence in patient and therapist-rated alliance predicts subsequent depressive symptoms in cognitive behavioral therapy for depression. Psychother. Res. 33, 719–728. doi: 10.1080/10503307.2022.2156304, PMID: 36525598 PMC10272287

[ref83] World Health Organization (2022). World mental health report: Transforming mental health for all. Available at: https://www.who.int/publications/i/item/9789240049338

[ref84] Zaitsoff S. Pullmer R. Cyr M. Aime H. (2015). The role of the therapeutic alliance in eating disorder treatment outcomes: a systematic review. Eat. Disord. 23, 99–114. doi: 10.1080/10640266.2014.964623, PMID: 25330409

